# Outbreak of Nontuberculous Mycobacteria Joint Prosthesis Infections, Oregon, USA, 2010–2016

**DOI:** 10.3201/eid2505.181687

**Published:** 2019-05

**Authors:** Genevieve L. Buser, Matthew R. Laidler, P. Maureen Cassidy, Heather Moulton-Meissner, Zintars G. Beldavs, Paul R. Cieslak

**Affiliations:** Oregon Health Authority, Portland, Oregon, USA (G.L. Buser, M.R. Laidler, P.M. Cassidy, Z.G. Beldavs, P.R. Cieslak);; Centers for Disease Control and Prevention, Atlanta, Georgia, USA (H. Moulton-Meissner)

**Keywords:** humans, Mycobacterium fortuitum, Mycobacterium goodii, nontuberculous mycobacteria, National Healthcare Safety Network, case–control studies, pulsed-field gel electrophoresis, surgical wound infection, bacteria, environment, knee joint, hip joint, arthroplasty, replacement, prostheses and implants, infection control, hair, soil, water, tuberculosis and other mycobacteria, Oregon, USA

## Abstract

We investigated a cluster of *Mycobacterium fortuitum* and *M. goodii* prosthetic joint surgical site infections occurring during 2010–2014. Cases were defined as culture-positive nontuberculous mycobacteria surgical site infections that had occurred within 1 year of joint replacement surgery performed on or after October 1, 2010. We identified 9 cases by case finding, chart review, interviews, surgical observations, matched case–control study, pulsed-field gel electrophoresis of isolates, and environmental investigation; 6 cases were diagnosed >90 days after surgery. Cases were associated with a surgical instrument vendor representative being in the operating room during surgery; other potential sources were ruled out. A tenth case occurred during 2016. This cluster of infections associated with a vendor reinforces that all personnel entering the operating suite should follow infection control guidelines; samples for mycobacterial culture should be collected early; and postoperative surveillance for <90 days can miss surgical site infections caused by slow-growing organisms requiring specialized cultures, like mycobacteria.

Rapidly growing environmental nontuberculous mycobacteria (NTM), including *Mycobacterium abscessus*, *M. chelonae*, and *M. fortuitum*, are uncommon but recognized causes of difficult-to-eradicate implant-associated infections (*1**–**3*). NTM prosthetic joint surgical site infections are associated with severe disease and require debridement, prosthesis excision, and prolonged administration of intravenous and broad spectrum antimicrobial drugs before prosthesis reimplantation (*3**,**4*). Such infections can result from inoculation of the surgical field or prosthesis during a surgical or medical procedure or from environmental contamination during the early postoperative period (*5*). A common potential source of infection, contaminated water, is not always identified despite thorough investigation (*6*). A less common source of implant-associated infections is transient NTM colonization of human hair and body sites (*7**,**8*). Rahav et al. showed how a hirsute surgeon, colonized with *M. jacuzzii* through frequent hot tub use, shed organisms during multiple breast implant surgeries (*7*). The investigators halted the outbreak by decolonizing the surgeon’s skin and hair and increasing operative infection control barriers.

In Oregon, USA, nonpulmonary, non–*Mycobacterium avium* complex NTM invasive isolates are uncommonly identified. During 2005–2006, a statewide laboratory survey of clinical microbiology isolates identified 28 *M. fortuitum–*group and 3 *M. goodii* isolates (*9*). During 2011, however, infection preventionists from 2 hospitals in the same region of Oregon reported 2 *M. goodii* prosthetic hip infections in close succession (cases 1 and 2; Table). A lack of additional cases that year precluded finding a statistical association with a common source. On January 1, 2014, extrapulmonary NTM isolates became reportable in Oregon (*10*). In May 2014, the same 2 hospitals and a third hospital in the region reported 4 *M. fortuitum* infections after prosthetic hip and knee implantations performed during July–December 2013 (cases 3, 4, 6, and 7). Root-cause analysis did not identify a common source. We launched an epidemiologic investigation to describe the cluster, determine the infection source, and guide control measures.

**Table:** Characteristics of 9 nontuberculous mycobacterial knee and hip prosthetic joint SSIs at multiple hospitals, Oregon, 2010–2014*

Case no.	Prosthesis type	Procedure year	SSI type	*Mycobacterium* species	Time to SSI, d†	SSI to culture, d‡	Time to culture, d§
1	Hip	2010	Unknown	*M. goodii*	157	0	157
2	Hip	2010	Organ space	*M. goodii*	35	35	70
3	Knee	2013	Deep incision	*M. fortuitum*	70	22	92
4	Knee	2013	Superficial incisional	*M. fortuitum*	85	0	85
5	Hip	2013	Organ space	*M. fortuitum*	78	54	132
6	Knee	2013	Organ space	*M. fortuitum*	69	34	103
7	Knee	2013	Organ space	*M. fortuitum*	69	30	99
8	Hip	2014	Suture abscess	*M. fortuitum*	86	14	100
9	Hip	2014	Organ space	*M. fortuitum*	83	1	84

*SSI identification date: National Health Safety Network event date, or chart review, or specimen culture collection date. Case 10 not included in analysis. SSI, surgical site infection. †From time of surgery to SSI identification date. Median 78 d. ‡From time of SSI identification to mycobacterial specimen culture collection date. Median 22 d. §From time of surgery to mycobacterial specimen culture collection date. Median 99 d.

## Methods

### Case Definition and Ascertainment

We reviewed surgical site infection cases reported by Oregon hospitals to the National Healthcare Safety Network (NHSN) during January 2009–May 2014 (*11*). A case was defined as a culture-positive NTM prosthetic joint surgical site infection within 1 year of joint replacement surgery performed in an Oregon patient on or after October 1, 2010. NHSN uses standardized definitions to harmonize healthcare-associated infection surveillance across US medical facilities. Oregon hospitals have been required to report knee prosthesis infections since 2009 and hip prosthesis infections since 2011. To identify cases not captured by NHSN surveillance, we queried healthcare providers within Oregon by using the Oregon Health Alert Network (*12*) and nationally by using *Epi-X* (*13*) and MedWatch (*14*).

### Chart Review

**Figure 1:**
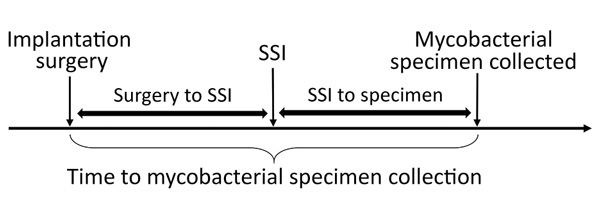
Time interval definitions used in investigation of mycobacterial prosthetic joint surgical site infections, Oregon, USA, 2010–2016. SSI, surgical site infection.

Case medical charts were reviewed by epidemiologists who used a modified version of the Centers for Disease Control and Prevention (CDC) healthcare-associated infection chart abstraction form to identify common exposures (e.g., hospital, surgery date, surgery type, prosthesis type and brand, surgeon, anesthesiologist, surgical assistants, vendor representatives, medications, surgical joint cement, and location to which patient was discharged) (*15**,**16*). We reviewed the following patient characteristics and exposures: municipal water source by address, postoperative wound care, and rehabilitation facility. To delineate the timeline of NTM surgical site infection identifications, we calculated 1) time from implantation surgery date to surgical site infection identification date, 2) time from surgical site infection identification date to mycobacterial culture specimen collection date, and 3) total time from implantation surgery date to mycobacterial culture specimen collection date (Figure 1). Surgical site infection identification date was defined as 1) NHSN event date; 2) date of clinical diagnosis as determined by chart review, if NHSN event date unavailable; or 3) specimen collection date, if information for the first 2 criteria was unavailable. Rapidly growing NTM are typically detected after 7–10 days of incubation on mycobacterial growth media.

### Interviews and Observations

To ascertain surgical instrument sterilization and implant inventory management practices, we interviewed infection preventionists in the 4 hospitals where case surgeries had been performed. We observed 3 joint prosthesis surgeries to assess general surgical procedures, generate hypotheses, and identify potential sources of surgical site contamination.

### Case–Control Study

To test associations between infection and exposures ascertained from chart review, including surgery records, we performed a 1:4 matched case–control study. We matched controls by hospital and type of prosthesis surgery (hip vs. knee) and restricted controls to surgeries performed within 6 months of the matched-case surgery date. Variables evaluated included surgeon, other operating room staff, time of day of surgery, patient age (>65 vs. <65 years), orthopedic device manufacturer, and surgeon orthopedic practice. We conducted analysis by using exact methods for matched data (SAS version 9.3, https://www.sas.com).

### Investigation of Clinical Specimens and Environment

Clinical isolates were available from all patients. Identification of isolates was confirmed by 16s rRNA gene sequencing. Pulsed-field gel electrophoresis (PFGE) was performed at CDC (National Center for Emerging and Zoonotic Infectious Diseases, Division of Healthcare Quality Promotion) as follows: chromosomal DNA was digested with the restriction endonuclease AseI (*17*), and restriction fragments were separated with a CHEF Mapper XA Pulsed-Field Electrophoresis System (Bio-Rad Laboratories, http://www.bio-rad.com) and analyzed for relatedness by BioNumerics software (Applied Maths, http://www.applied-maths.com). Similarity of PFGE patterns was based on Dice coefficients, and a dendrogram was built by using the unweighted pair group method. The Tenover criteria were used to classify comparisons of patient isolate PFGE patterns as indistinguishable (100% similarity), closely related (1–3-band difference), possibly related (4–6 band difference), or unrelated (>7 band difference) (*18*). Environmental investigation was based on investigation results, findings from published NTM investigations (*7**,**8*), and expert consultation with CDC.

## Results

### Descriptive Epidemiology

In addition to the 2 cases of unknown exposure source from 2011 and the 4 cases reported during May 2014, review of NHSN data identified a previously unreported case of *M. fortuitum* prosthetic hip infection from the same Oregon healthcare region during 2013 (case 5). Queries of the Oregon Health Alert Network, *Epi-X*, and MedWatch did not identify additional cases or prosthesis recalls in Oregon or other US states. Cases 8 and 9 occurred during the investigation (Table).

**Figure 2:**
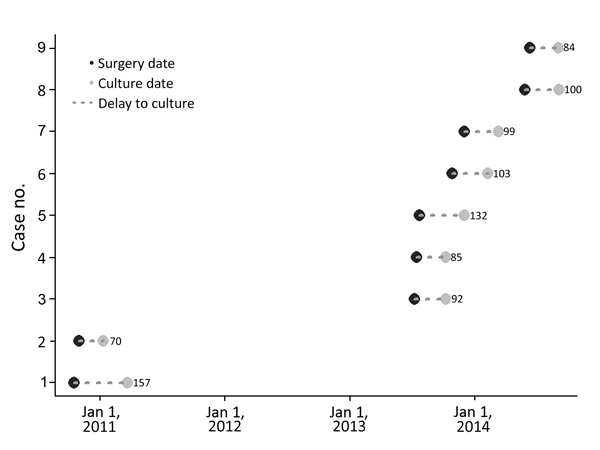
Time intervals between knee and hip prosthetic joint surgery to collection of surgical site mycobacterial cultures yielding related nontuberculous mycobacteria, multiple hospitals, Oregon, 2010–2014 (n = 9). Numbers indicate total number of days from surgery (black dots) to culture collection (gray dots). Case 10 is not included.

We identified a total of 7 *M. fortuitum* and 2 *M. goodii* surgical site infections involving 4 knee and 5 hip prostheses; 5 were deep organ space infections. Patients were 46–79 (median 68) years of age, and 5 were female and 4 male; none had signs of infection at the time of prosthesis placement. Surgeries were performed at 4 hospitals by 6 different surgeons. Surgeries were performed during October 2010–June 2014; corresponding infection onsets occurred during January 2011–September 2014. Cases clustered in July (3 cases) and October (3 cases); others occurred in May, June, November, and December (1 case each) (Figure 2). Time from implantation surgery to surgical site infection identification was 35–157 (median 78) days (Table), from surgical site infection identification to mycobacterial specimen collection was 0–54 (median 22) days, and from implantation to specimen collection for mycobacterial culture was 70–157 (median 99) days. For 6 (67%) patients, >90 days elapsed between implantation and collection of the specimen for mycobacterial culture that yielded NTM.

### Case–Control Study 

NTM surgical site infections were significantly associated with implantation of a brand A prosthesis (unadjusted matched odds ratio [mOR] 27.7, 95% CI 5.3–∞; p = 0.0002), with surgeon A (mOR 15.4, 95% CI 2.3–∞; p = 0.016), and with the presence of person A (a brand A vendor) in the operating room during surgery (mOR 32.4, 95% CI 6.3–∞; p = 0.0001). All case-patients had brand A prostheses implanted, but the devices and lot numbers differed. Surgeon A was present at only 3 of the 9 surgeries and did not perform any surgeries at 2 of the hospitals. Person A was the brand A prosthesis vendor representative at all 4 hospitals, and operating room records documented person A’s presence at all surgeries except that of case 5 (8 [89%] of 9 patients).

### Interviews and Observations

During 3 observed surgeries (not attended by person A), lapses in operating room infection control standards by other prosthesis vendor representatives were observed. Lapses included uncovered arms, uncovered hair and beards, and breaches of the vertical sterile field boundary over the surgical instrument table.

Infection preventionists reported that vendors regularly transport loaner instruments for prosthetic surgeries between hospitals; hospital policies require, and infection preventionists confirmed, that before surgical use such instruments are reprocessed according to each facility’s internal standards. Person A reported similar loaner instrument practices and did not report using or transporting other undocumented tools or instruments into the surgeries involved in the outbreak reported here. None of the 4 hospitals reported failures of biological indicators of sterilization during the period investigated.

### Clinical Specimens

**Figure 3:**
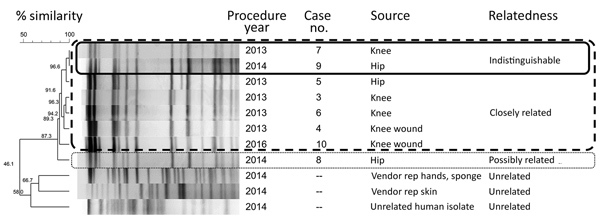
Dendrogram of 8 *Mycobacterium fortuitum* isolates associated with prosthetic joint surgical site infections, multiple hospitals, Oregon, 2013–2016. Boxes indicate group relatedness according to pulsed-field gel electrophoresis: solid lines, indistinguishable (no band difference); dashed lines, closely related (1–3 band difference); dotted lines, possibly related (4–6 band difference). Differences of >7 bands indicate not related. Rep, representative.

PFGE indicated that *M. goodii* isolates from cases 1 and 2 were closely related. *M. fortuitum* isolates from cases 7 and 9 were indistinguishable (100% similarity). Isolates from cases 3, 4, 5, and 6 were closely related to isolates from cases 7 and 9 (1–3 band differences, >89% similarity); and the isolate from case 8 was possibly related to isolates from cases 3, 4, 5, 6, 7, and 9 (4–6 band differences, >87% similarity) (Figure 3).

### Environmental Specimens

On the basis of the above findings, on 1 day we collected environmental and human samples from the home of person A. Environmental samples were collected from person A’s hot tub water, filter, cover, and headrest; bathroom showerhead and faucet aerators; washing machine; cell phone; and touch computer screen. Human samples from person A included skin; scalp hair; eyebrows; facial hair; and swab specimens of nares, ears, and hands. Specimens were shipped directly to CDC for immediate processing and culture. Acid-fast isolates were identified by 16s rRNA sequencing. On the same day as sample collection, a registered environmental health specialist reviewed person A’s hot tub maintenance processes on site.

In total, we collected 16 environmental samples from person A’s residence and 9 samples directly from person A. *M. fortuitum* was isolated from person A’s hands, but the strain was genetically unrelated to the outbreak isolates (Figure 3). Culture of environmental samples yielded heavy growth of non-NTM bacteria, and none yielded slow-growing NTM. Person A reported doing yard work and using a personal outdoor hot tub daily; however, hot tub water chlorination and pH were not routinely monitored. At the time of specimen collection, hot tub water pH was >8.0 (optimal pH for chlorinated tubs is 7.4–7.6) (*19*).

### Infection Control Response

When cases 8 and 9 were reported during the investigation, the Oregon Health Authority Public Health Division recommended immediate sequestration of remaining unused, packaged brand A products and that person A complete infection control training consistent with guidance from the Association of periOperative Registered Nurses before returning to the operating suite (*20*). Hospitals involved in the outbreak required infection control training for all persons entering the operating rooms, including vendors. Operating room audits were increased to monitor guideline compliance of all staff. Person A was advised to use hot tub disinfection and chlorination practices consistent with the manufacturer’s recommendations. Person A returned to work 2 months later.

### Follow-Up

After 28 months of no reported outbreak cases, hospital B identified an *M. fortuitum* superficial surgical site infection 37 days after a knee prosthesis replacement surgery in which a brand B prosthetic was used (case 10). The new isolate was closely related to that of the outbreak by PFGE (3-band difference), suggesting epidemiologic linkage to the outbreak cases (Figure 3). Surgeons other than those involved in the original outbreak series performed the surgery; however, the vendor present was person A.

## Discussion

Although we identified no common intraoperative water or fomite source, this uncommon geotemporal and PFGE-linked cluster of NTM prosthetic joint surgical site infections was statistically associated with the presence of a non–hospital employee (the prosthetic joint vendor, person A). Although the outbreak strain was not isolated from person A, epidemiologic evidence suggests repeated introduction of the NTM outbreak strains into multiple knee and hip prosthetic joint surgeries over 6 years and across 4 facilities. Other reports have suggested the possibility of NTM human colonization—made possible by repeated exposure to hot tub water, which, when coupled with lapses in infection control practices, leads to the entry of NTM via squamous epithelial cells or body hair shed into the sterile field (*7**,**8**,**21**,**22*). Other hypotheses include an as-yet unidentified instrument fomite associated with person A, for which the usual hospital policy for loaner instrument sterilization was circumvented (*23*). However, sterilization process failures were not identified at any of the facilities, making simultaneous contamination of an instrument or instruments at 4 different hospitals implausible.

Most NTM surgical site infection investigations have focused on contamination by nonsterile water but did not identify a contaminated water source (*4**,**6**,**24*). Modern operating suites are constructed to avoid this known risk. Of note, isolation of an NTM strain from person A’s hands supports the biological plausibility of transmission via healthcare worker contact. Persistent hand and skin colonization with NTM organisms could occur after contact with soil and water sources and other outdoor activities. Hot tubs are a known source of NTM infections, including *M. fortuitum* infections*,* presumably via water droplets (*25*). There is evidence that NTM can be transmitted via colonized persons, from contaminated water sources such as hot tubs, to the sterile surgical field, leading to surgical site infections (*7**,**8*). Of note, the pH of person A’s hot tub water at the time of the environmental investigation was >8, which is ineffective for disinfection (*19*).

Because NTM are widespread and colonization can occur at any time, adherence to perioperative infection control guidelines by all operating suite personnel, including vendors, is necessary (*20**,**23*). Vendors are active members of the operating team and should be required to complete infection control training that meets hospital standards for other operating room personnel. Hospitals should consider sharing surgical site infection rates with product vendors to highlight their accountability in preventing surgical site infections. Even without direct patient contact, vendors could be vectors that transmit pathogens into the operating room environment via rectal or nasal colonization, spread shed skin and hair cells via air currents, or transport contaminated fomites into the operating suite (*26**,**27*). 

The unusual organisms involved enabled detection of this outbreak of prosthetic joint–associated surgical site infection and the subsequent search for a source. If the pathogens had been more common surgical site infection–associated skin colonizers, such as coagulase-negative staphylococci, this outbreak could have remained undetected and persisted indefinitely. Indeed, it is possible that more typical surgical site infections stem from the operating room presence of vendors or others for whom infection prevention standards are overlooked.

Identifying and linking NTM surgical site infections is challenging for multiple reasons. Surgical teams, including vendors, can rotate among multiple hospitals, decreasing the likelihood that epidemiologically related cases will be recognized as such. Although culture on mycobacteria-specific media improves yield, mycobacterial cultures are often not sent until after the initial bacterial cultures are negative, resulting in an apparent delay in identification of NTM surgical site infection; in 6 of 9 cases in this outbreak, mycobacteria were first grown from specimens obtained >90 days postoperatively. In 2015, when NHSN shortened the postoperative infection surveillance period after knee and hip prosthesis implantation surgeries from 365 to 90 days, surveillance may have shifted toward detection of acute bacterial infections and away from detection of slow-growing NTM infections, which require special culture media.

Since January 2014, Oregon has required laboratories to report extrapulmonary NTM infections, and cases are routinely investigated by public health officials (*10*). From January 2014 through December 2017, a total of 150 cases were reported, including 2 clusters: *M. fortuitum* infections associated with abdominoplasty in an ambulatory surgery center and *M. haemophilum* infections associated with using water from a water cooler to dilute tattoo ink before subcutaneous injection (*28**,**29*). To assist surveillance in other states and territories, in 2017 the Council for State and Territorial Epidemiologists adopted a standardized case definition for extrapulmonary NTM infections (*30*). In the presence of a 90-day postoperative surveillance period, state surveillance of nonpulmonary NTM infections may provide a method for identifying these uncommon but medically complex surgical site infections.

This investigation has several limitations. Cases might have been missed despite NHSN surveillance before nonpulmonary NTM infections became reportable in Oregon in January 2014, because NHSN definitions do not include superficial surgical site infections identified >30 days after surgery (e.g., case 10). The likelihood of missing cases during the investigation was small because of extensive case finding by multiple methods, including mandatory reporting by Oregon laboratories and direct communication with infection preventionists. Although early analysis indicated that the presence of surgeon A was significant, surgeon A did not have operating privileges at 2 of the hospitals and therefore could not have been a source of exposure for those cases. Although loaner instrument storage is not regulated outside of the hospital, all hospitals had strict reprocessing policies in place for cleaning and sterilizing trays received. In the case–control analysis, the association between person A and surgical site infection was confounded by brand A; no NTM surgical site infections linked to the brand were reported elsewhere in Oregon or nationally, suggesting that brand A per se was not a factor in the outbreak. In addition, case 10 occurred when person A was a vendor for a different prosthetic company. Although unidentified fomites associated with person A can never be summarily excluded without a direct microbiological link from an environmental source via person A to the surgical site, we believe that the totality of the epidemiologic and observational evidence leaves the most likely source of these infections to be human colonization.

All persons, including vendors, who enter operating suites should recognize that environmental exposures (e.g., hot tub bathing, gardening, field work) can colonize their hands, skin, and hair with infectious organisms such as NTM, which can then cause surgical site infections. All personnel and vendors in the operating suite should adhere to published guidelines about perioperative infection prevention, surgical attire, and environmental controls (*20*,*23*) and should be required to undergo annual infection control training. Infection preventionists should regularly observe operating room staff to verify compliance and to promptly apply any necessary corrective measures. When evaluating device-associated surgical site infections, clinicians should obtain mycobacterial along with bacterial and fungal cultures. Extrapulmonary NTM infection surveillance is an option for public health jurisdictions to increase detection of these slow-moving, but morbid, outbreaks. Finally, specialized measures like environmental and human sampling to isolate fastidious NTM organisms are recommended during investigations and could reveal the elusive microbiological link.
